# Double right coronary artery with fistula to the right atrium and mitral insufficiency: A case report

**DOI:** 10.1016/j.ijscr.2022.107386

**Published:** 2022-07-06

**Authors:** Mohammad Nour Aldeen Koukash, Marah Hamad, Adeeb Makhlouf, Ameer Kakaje, Bayan Alsaid

**Affiliations:** a Faculty of medicine, Damascus University, Damascus, Syria; b Department of cardiac surgery, Alassad University Hospital, Damascus University, Damascus, Syria; c Department of General Surgery, Alassad University Hospital, Damascus University, Damascus, Syria; d Laboratory of Anatomy, Faculty of Medicine, Damascus University, Damascus, Syria; e University Hospital Geelong, Barwon Health, Victoria, Australia

**Keywords:** CA, Coronary artery, CABG, Coronary artery bypass grafting, CAF, Coronary artery fistula, Cx, Circumflex artery, ECG, Electrocardiogram, LAO, Left anterior oblique, MDCT, Multidetector-row computed tomography, RA, Right atrium, RCA, Right coronary artery, RV, Right ventricle, Angina, Angiogram, Coronary artery anomalies, Double right coronary artery, Fistula, Mitral regurgitation

## Abstract

**Introduction and importance:**

Coronary artery abnormalities are uncommon and mostly asymptomatic. The combination of double right coronary artery (RCA) with a fistula and valvar deformity is very rarely reported in the literature. However, it is important to identify these deformities as they have relatively high complication rates.

**Presentations of case:**

A 47-year-old male came with chest pain that radiated to the lower jaw. ECG showed equivalent changes. Blood tests including troponin were within normal range. However, echocardiogram showed a severe mitral valve regurgitation with anterior leaflet prolapse and hypokinesia of the ventricular wall. Coronary angiogram showed a double RCA with a complete block in the main RCA and a fistula to the right atrium (RA). The left coronary system showed atherosclerosis in left anterior descending artery (LAD) and circumflex artery (CX). Surgical treatment, including the repair of the RCA-RA fistula, replacement of mitral valve and coronary artery bypass grafting (CABG) were performed. The patient had no complications in the follow-ups.

**Discussion:**

Coronary fistulas may be congenital or acquired malformations. Their treatment depends on the symptoms, origin, size and the receiving chamber. Furthermore, double RCA is debatable whether the rate of atherosclerosis and other cardiac abnormalities are increased with this anomaly. The surgeon must keep in mind these rare anomalies before cardiac operations.

**Conclusion:**

Double RCA might accompany other deformities which are important to detect before intervention. More studies are required to decrease complications and have better outcomes.

## Highlights

• Double RCA is rare and may co-exist with other heart pathologies.
• Its management depends on site, size, associated symptoms and concomitant pathology.
• It may mimic coronary artery dissection and predispose to arteriosclerosis.

## 1 Introduction

Coronary artery (CA) anomalies are uncommon and present in 0.2–1.4 % of the population [1]. Double right coronary artery (RCA) is one of these anomalies with an incidence of 0.01–0.46 % [2]. Only rare cases were reported when double RCA accompanied other deformities [2].

We present a rare combination of double RCA with fistula to the right atrium (RA) with severe mitral regurgitation with severe anterior leaflet prolapse. Such a combination is rarely reported in the literature despite affecting the outcomes and having high complication rates.

## 2 Case presentation

A 47-year-old male presented with intermittent chest pain on exertion that radiated to the lower jaw and decrease exercise tolerance for 20 days. The patient never had similar symptoms before. The patient had history of smoking 12 pack/year with no other significant medical or surgical history. The patient had a family history of hypertension, diabetes, and ischemic heart disease.

No fevers were recorded. The electrocardiogram (ECG) on presentation showed sinus tachycardia of 101 beats/min, and an isolated ST depression in lead II. Blood investigations including troponin and creatine kinase-MB were within normal range. Inflammatory markers and blood cultures were negative.

**Fig. 1:**
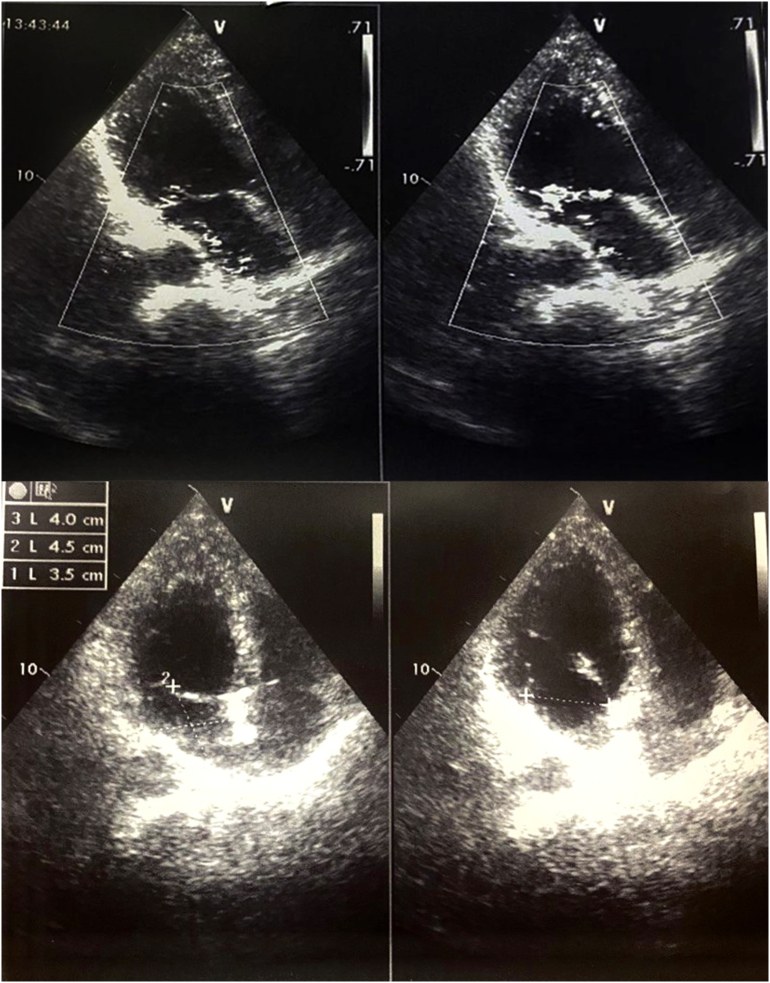
Showing transthoracic echocardiogram which demonstrated severe al regurgitation and anterior leaflet A2 prolapse from ischemia and degenerative changes. The annulus measuring 35 mm (normal from 28 to 32).

Inpatient echocardiogram demonstrated hypokinesia in the anterolateral wall of the left ventricle with an ejection fraction of 53 %, fractional shortening of 27 %, mitral valve anterior leaflet prolapse A2 with severe regurgitation (3–4/4) that was believed to be from ischemia and degeneration, and moderate pulmonary hypertension (Fig. 1).

**Fig. 2:**
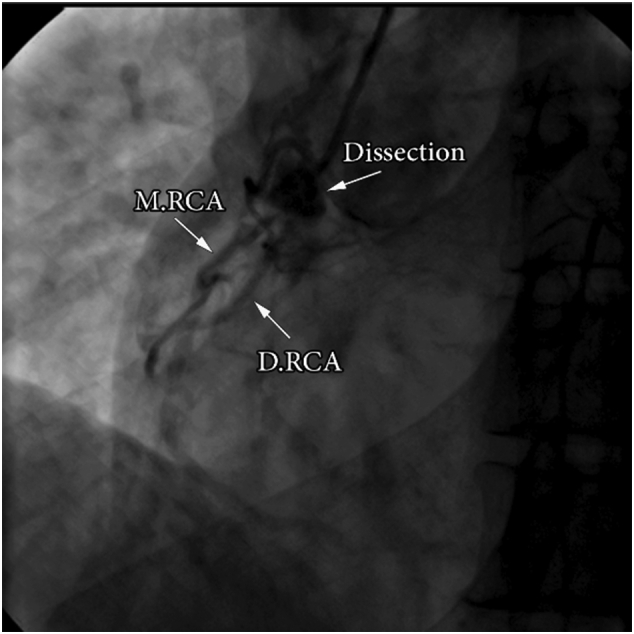
Coronary angiography in the Left anterior oblique (LAO) caudal view demonstrated double right coronary artery: main RCA (M.RCA), double RCA (D.RCA) and the dissection in the proximal portion of the main RCA.

**Fig. 3:**
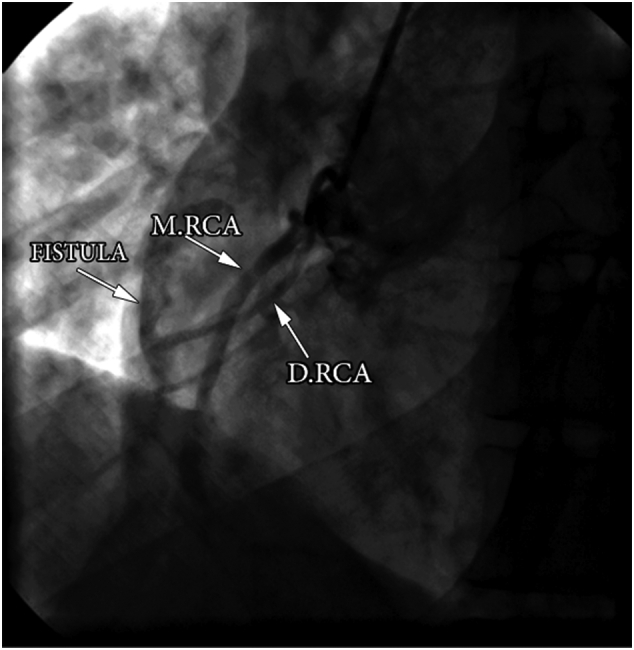
Coronary angiography in the LAO caudal view demonstrated fistula from the double RCA (D.RCA) to the RA and main RCA (M.RCA).

Coronary angiography was conducted using (JL4\JR4) diagnostic catheter, showing a full occlusion in the proximal portion of the left descending artery (LAD) with late retrograde filling. It also showed stenosis in the circumflex (CX) artery, approximately 60 % in the first segment. Injection contrast into the right sinus of Valsalva revealed double RCAs, the main RCA was completely blocked including the origin with right-left retrograde flow. Leaking opacification at the entrance of the double RCA to the RA demonstrated a coronary artery fistula (CAF) (Fig. 2, Fig. 3). A dissection occurred in the first segment of the main RCA during the injection in the right sinus of Valsalva (Fig. 2), which led to ventricular fibrillation. The procedure was then ceased, and the patient was successfully resuscitated. Then, the patient was transferred to the intensive care unit where a multi-slice computed tomography was performed which confirmed the absence of aortic root dissection and the dissection in first segment of the main RCA was stable.

**Fig. 4:**
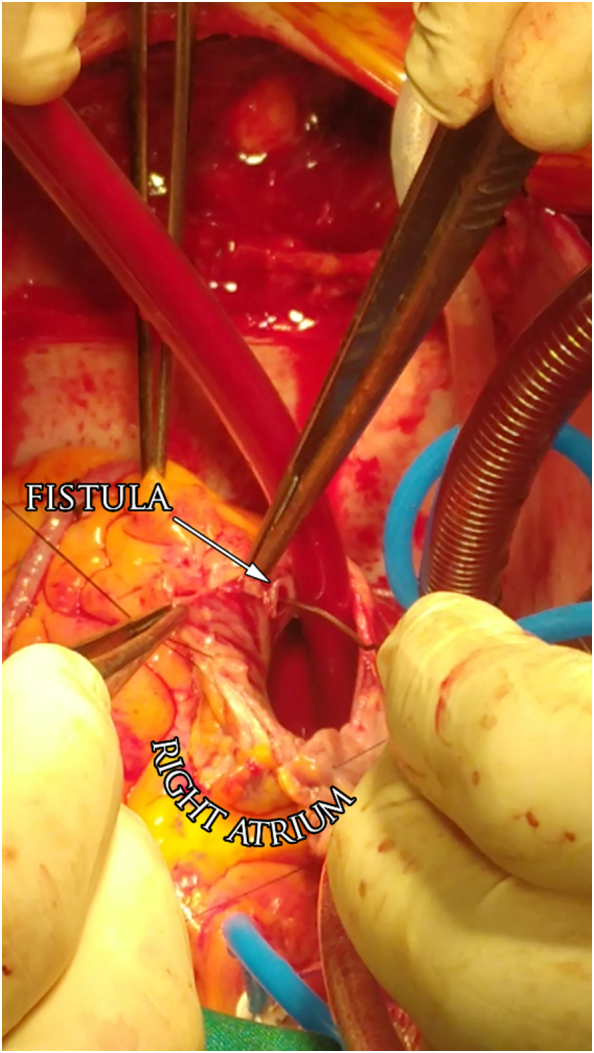
Image during surgical intervention illustrated the orifice of the fistula in the right atrium.

An open-heart surgery to replace the mitral valve and to perform CABG was later successfully performed. The surgery was delayed for 12 days to treat dental abscesses and for the patient to fully recover from the angiogram. During the surgery, a duplicate RCA was found deep in the cardiac fatty tissue and the surgeon was unable to fully dissect the adipose tissue surrounding the RCA to protect cardiac structures. The RA was opened, and the fistula was found between the pectinate muscles (Fig. 4). The fistula was closed with one stitch “Figure-eight”. The A2 prolapse of the anterior leaflet of the mitral valve was managed by replacing the mitral valve with a Medtronic mechanical valve. The valve was not repaired due to the valvular defect being from a mixed aetiology, unavailability of special cords to repair the valve, the defect being in A2 leaflet, not A1, and unavailability of transoesophageal echocardiogram which is necessary to check the function of the valve after repair. Coronary artery bypass grafting (CABG) was performed using a saphenous vein graft connected to the distal right coronary artery and left obtuse marginal artery and left internal thoracic artery graft connecting to the left anterior descending artery. Surgery was successfully performed without complications. Six-month follow-ups showed symptoms resolve with no complications.

## 3 Discussion

Double RCA might arise from either a single ostium and then split into two branches or from different ostia in the right sinus of Valsalva [3].

CAFs are an abnormal connection between a coronary artery and a heart chamber or any segment of the pulmonary or systemic circulation. They could be congenital or developed after a trauma, infection, or after medical procedures [1], [4]. CAFs have incidence of 0.67 % of total cardiac patients [5].

CAFs of the RCA are the most common, comprising 50 to 60 % of cases and the right ventricle (RV) is the most common chambers for these fistulas to connect to (45 %), followed by the RA (25 %) [4], [6]. In our case, the fistula connected to the RA.

Most double RCA cases are asymptomatic. However, some studies noticed an increased risk of atherosclerosis, aneurysms, and valvular deformities while others debate that there was no increase [2], [4], [5], [7]. In our case, double RCA was associated with atherosclerosis in the left and right coronary system.

In comparison, Small CAFs are usually asymptomatic and can close spontaneously. However, large CAFs can lead to clinical consequences such as cardiac chamber enlargement or myocardial infarction [8].

Double RCA and CAFs diagnosis can be made by angiography, echocardiography and multidetector-row computed tomography (MDCT) [4], [9]. Double RCA is not easy to find by the surgeon during the operation. The diagnosis of similar anomalies if suspected should be using multiple methods such as direct visualization in surgery, coronary angiography and other procedures [7]. This is particularly important as double RCA during the angiogram might mimic a coronary dissection [2]. Furthermore, the highly angulated anatomy of the RCAs and the small size of these arteries which could be also stenotic will make the procedure more difficult to conduct and have higher complications rate [2]. Only few cases were reported were double RCAs accompanied structural deformity such as aortic and mitral valve abnormalities [2].

This work has been made per The SCARE 2020 guidelines to ensure high-quality reporting [10].

## 4 Conclusion

Double RCA is rare and may co-exist with other pathologies such as fistulae. Management strategies should include identifying and treating concomitant pathology. There is a need for more reporting and further research on the subject.

## Ethics approval and consent to participate

Informed consent was taken for this research. Our study ethical aspects were reviewed and approved by Damascus University deanship, Damascus, Syria.

## Consent for publication

Written informed consent was obtained from the patient for publication of this case report and accompanying images. A copy of the written consent is available for review by the Editor-in-Chief of this journal on request.

## Funding

No funding was received for this study.

## Guarantor

Dr. Bayan Alsaid is the guarantor for the images and the case.

## Provenance and peer review

Not commissioned, externally peer-reviewed.

## Declaration of competing interest

We have no conflict of interest to declare.

## References

[1] Jacobs M.L., Mavroudis C. (2010). Anomalies of the coronary arteries: nomenclature and classification. Cardiol. Young.

[2] Chien T.M., Chen C.W., Chen H.M., Lee C.S., Lin C.C., Chen Y.F. (2014). Double right coronary artery and its clinical implications. Cardiol. Young.

[3] Sari I., Kizilkan N., Sucu M., Davutoglu V., Ozer O., Soydinc S. (2008). Double right coronary artery: report of two cases and review of the literature. Int. J. Cardiol..

[4] Kidawa M., Peruga J.Z., Foryś J., Krzemińska-Pakuła M., Kasprzak J.D. (2009). Acute coronary syndrome or steal phenomenon - a case of right coronary to right ventricle fistula. Kardiol. Pol..

[5] Resatoglu A.G., Elnur E.E., Yener N., Elhassan H., Yener A. (2005). Double right coronary artery; fistula and atherosclerosis: rare combination. Jpn. J. Thorac. Cardiovasc. Surg.ery : official publication of the Japanese Association for Thoracic Surgery = Nihon Kyobu Geka Gakkai zasshi.

[6] Knippel M., Ravizza P., Gullace G., Bana G., Savoia M., Locatelli V. (1982). An unusual case of congenital double coronary arteriovenous fistula. Chest.

[7] Erbagci H., Davutoglu V., Turkmen S., Kizilkan N., Gumusburun E. (2006). Double right coronary artery: review of literature. Int.J.Cardiovasc.Imaging.

[8] Al-Hijji M., El Sabbagh A., El Hajj S., AlKhouli M., El Sabawi B., Cabalka A. (2021). Coronary artery fistulas: indications,techniques, outcomes, and complications of transcatheter fistula closure. JACC Cardiovasc. Interv..

[9] Graidis C., Dimitriadis D., Karasavvidis V., Dimitriadis G., Argyropoulou E., Economou F. (2015). Prevalence and characteristics of coronary artery anomalies in an adult population undergoing multidetector-row computed tomography for the evaluation of coronary artery disease. BMC Cardiovasc. Disord..

[10] Agha R.A., Franchi T., Sohrabi C., Mathew G., Kerwan A., Thoma A. (2020). The SCARE 2020 guideline: updating consensus Surgical CAse REport (SCARE) guidelines. Int. J. Surg..

